# Risk Factors for Gambling Problems on Online Electronic Gaming Machines, Race Betting and Sports Betting

**DOI:** 10.3389/fpsyg.2017.00779

**Published:** 2017-05-15

**Authors:** Nerilee Hing, Alex M. Russell, Matthew Browne

**Affiliations:** Experimental Gambling Research Laboratory, School of Health, Medical and Applied Sciences, Central Queensland University, BundabergQLD, Australia

**Keywords:** gambling, problem gambling, risk factors, internet gambling, online gambling, gambling disorder, interventions

## Abstract

Growth of Internet gambling has fuelled concerns about its contribution to gambling problems. However, most online gamblers also gamble on land-based forms, which may be the source of problems for some. Studies therefore need to identify the problematic mode of gambling (online or offline) to identify those with an online gambling problem. Identifying most problematic form of online gambling (e.g., EGMs, race betting, sports betting) would also enable a more accurate examination of gambling problems attributable to a specific online gambling form. This study pursued this approach, aiming to: (1) determine demographic, behavioral and psychological risk factors for gambling problems on online EGMs, online sports betting and online race betting; (2) compare the characteristics of problematic online gamblers on each of these online forms. An online survey of 4,594 Australian gamblers measured gambling behavior, most problematic mode and form of gambling, gambling attitudes, psychological distress, substance use, help-seeking, demographics and problem gambling status. Problem/moderate risk gamblers nominating an online mode of gambling as their most problematic, and identifying EGMs (*n* = 98), race betting (*n* = 291) or sports betting (*n* = 181) as their most problematic gambling form, were compared to non-problem/low risk gamblers who had gambled online on these forms in the previous 12 months (*n* = 64, 1145 and 1213 respectively), using bivariate analyses and then logistic regressions. Problem/moderate risk gamblers on each of these online forms were then compared. Risk factors for online EGM gambling were: more frequent play on online EGMs, substance use when gambling, and higher psychological distress. Risk factors for online sports betting were being male, younger, lower income, born outside of Australia, speaking a language other than English, more frequent sports betting, higher psychological distress, and more negative attitudes toward gambling. Risk factors for online race betting comprised being male, younger, speaking a language other than English, more frequent race betting, engaging in more gambling forms, self-reporting as semi-professional/professional gambler, illicit drug use whilst gambling, and more negative attitude toward gambling. These findings can inform improved interventions tailored to the specific characteristics of high risk gamblers on each of these online activities.

## Introduction

Participation in online gambling continues to increase in tandem with its deregulation, prolific advertising, the widespread uptake of computer and mobile technologies, and increased availability of high speed internet access (Wood and Williams, 2011; Gainsbury, 2012; Hing et al., 2014a). These developments have fuelled concerns that online gambling contributes substantially to gambling problems, prompting research into online problem gamblers, including their characteristics, prevalence, and associated risk factors (Wood and Williams, 2007, 2009; Wardle et al., 2011; Gainsbury et al., 2013; Hing et al., 2014b). While these studies have provided insights into the characteristics and behaviors of problem gamblers who gamble online, they have typically not accounted for an important issue that can confound such analyses. This is that not all problem gamblers who engage in online gambling have a gambling problem related to their online gambling. In fact, most online gamblers also gamble on land-based forms (Wardle et al., 2011; Gainsbury et al., 2015a) which may be the main source of problems for some. Thus, to automatically attribute gambling problems amongst online gamblers to their use of online modes of gambling is inaccurate and overestimates the impact of Internet technologies on problem gambling.

A second issue potentially confounding an accurate understanding of problem online gambling is that measures of problem gambling, such as the PGSI (Ferris and Wynne, 2001), DSM (American Psychiatric Association, 2000, 2013) and the South Oaks Gambling Screen (Lesieur and Blume, 1987), do not distinguish the gambling form(s) causing problems. Both of these issues may lead to erroneous conclusions. For example, based on the above assumptions, an individual with a gambling problem related to land-based electronic gaming machines (EGMs) and who occasionally purchases an online lottery ticket (and is therefore classified as an online gambler) may be wrongly assumed to have problems with online lottery gambling. Both online gambling and lottery gambling would be incorrectly implicated in this example, based on the usual categorisation of problem online gamblers and the inability of traditional measures of problem gambling to distinguish the gambling form causing problems.

Studies need to identify the problematic mode of gambling (online or offline) and problematic form of gambling (e.g., EGMs, race betting, sports betting) to be able to more accurately characterize those with a gambling problem attributable to a specific online gambling form. This study pursues this approach and aims to: (1) determine demographic, behavioral and psychological risk factors for gambling problems on online EGMs, online sports betting and online race betting; and (2) compare the characteristics of problematic online gamblers on each of these online forms. Understanding risk factors is important to inform improved targeting of harm minimisation and other public health measures for Internet gambling. Further, identifying risk factors for each online gambling form will enable additional tailoring of these measures to high-risk consumers who engage in each of these activities. Literature on the characteristics of online gamblers and online problem gamblers, along with associated risk factors, is now briefly reviewed to contextualize this study.

### Characteristics of Online Gamblers

Several studies have compared online gamblers to offline gamblers (Griffiths et al., 2009, 2011; Wood and Williams, 2009; Gainsbury et al., 2012, 2015b). These analyses have classified online gamblers as those who have gambled at least once in the past year using an Internet mode of gambling, with the remaining gamblers classified as offline gamblers. These analyses therefore distinguish non-Internet gamblers from individuals who have gambled online. This latter group may include online-only gamblers, those who gamble mainly online and those who gamble mainly offline, including one-time-only online gamblers. Thus, individuals classified as online gamblers can vary widely in their proportionate engagement with online modes of gambling. Despite this heterogeneity, a reasonably consistent profile of online gamblers has emerged. Compared to offline gamblers, online gamblers are more likely to be male, younger, more highly educated, have higher incomes, engage in a greater number of gambling activities, and have higher problem gambling rates. (Griffiths et al., 2009; Wood and Williams, 2009; Svensson and Romild, 2011; Gainsbury et al., 2015b). More fine-grained analyses categorizing online gamblers into online-only, offline-only and mixed-mode gamblers have generally found these characteristics to be more pronounced amongst mixed-mode gamblers (Wardle et al., 2011; Gainsbury et al., 2015a).

### Characteristics of Problem Online Gamblers and Associated Risk Factors

Research has also compared problem online gamblers to non-problem online gamblers to determine risk factors for gambling problems amongst Internet gamblers. Compared to non-problem online gamblers, problem online gamblers tend to be male, younger, to gamble on a wider range of activities, to have higher gambling expenditure, to hold more erroneous gambling beliefs, and to hold more negative attitudes toward gambling (Wood and Williams, 2007, 2009; Gainsbury et al., 2015c). Other studies have compared online problem gamblers to offline problem gamblers, in attempting to isolate distinctive risk factors associated with online modes of gambling. Amongst problem and moderate risk gamblers, Gainsbury et al. (2013) found that those who gambled online were more likely to be younger, engage in more types of gambling activities, and to bet on sports. In a clinical sample of problem gamblers, those who gambled online were found to have higher educational levels, socio-economic status, gambling expenditure and gambling debts, with no differences in clinical, psychopathological and personality characteristics, compared to offline problem gamblers (Jiménez-Murcia et al., 2011).

While the above two types of comparisons are legitimate and informative, confusion arises when results are interpreted as meaning that online gambling is necessarily the source of gambling problems amongst those categorized as problem online gamblers. Indeed, the relatively high problem gambling rates found amongst online gamblers (Petry and Weinstock, 2007; Wood and Williams, 2007, 2011; Griffiths et al., 2009; Hing et al., 2014a) have often been inferred as meaning that online gambling is riskier than land-based gambling. However, as noted above, offline modes may be the source of gambling problems for a proportion of this group. Indeed, Wood and Williams (2007) found that a preference for non-Internet gambling was one predictor of problematic gambling amongst a sample of 1,920 Internet gamblers. They therefore speculated that online gambling may not necessarily facilitate problem gambling, but that problem gamblers may instead be drawn to online gambling, or more generally, multiple modes of product delivery. This emphasizes the need to distinguish most problematic gambling mode amongst online problem gamblers in order to correctly assess the role of Internet modes of access in problem gambling.

To our knowledge, only one study has analyzed the characteristics of problem gamblers whose gambling problems relate specifically to online gambling (Hing et al., 2015b). Amongst a convenience sample of 620 problem gamblers, 46% nominated an Internet gambling mode as their most problematic; with access being made either via computers, mobile phones, tablets, or interactive television. These problem online gamblers were significantly more likely than problem offline gamblers to be male, younger, have lower psychological distress, experience problems with sports and race betting, spend more time and money on these gambling forms, have lower problem gambling severity and lower rates of help-seeking. While that study more accurately categorized problem online and offline gamblers and provided useful insights into how they differ, it did not examine factors that increase the risk of transitioning from a non-problem online gambler to a problem online gambler. It also did not examine risk factors for different forms of online gambling, as this current study will do.

In summary, most studies of online problem gamblers have not determined whether their gambling problem is specifically related to an Internet mode of gambling. These analyses therefore include online gamblers with offline gambling problems. This lack of distinction of most problematic gambling mode amongst dual-mode gamblers means that risk factors for online gambling remain uncertain. Further, these studies have not distinguished which form of online gambling is most problematic. They have therefore been unable to identify risk factors for specific forms of online gambling. This study seeks to overcome these issues by conducting analyses comparing those who attribute their problems to each of three forms of online gambling (EGMs, sports betting, race betting) to those who also gamble online on those forms and have not experienced problems, and then comparing the groups who attribute their problems to these three online forms to each other. These analyses are the first to specifically study the characteristics and risk factors of gamblers whose problems develop in each of these online gambling forms. Because of the early stage of this avenue of research, the current research is considered exploratory and no specific hypotheses are presented Similar to previous analyses of risk factors for different types and modes of gambling (e.g., Wood and Williams, 2009; Gainsbury et al., 2015c; Hing et al., 2016e), a range of demographic, behavioral and psychological factors are included, as identified below. The findings can inform improved interventions tailored to the specific characteristics of high-risk gamblers on each of these online gambling activities.

## Materials and Methods

### Participants and Recruitment

A total of 4,594 eligible respondents completed an online survey which targeted Australian adults who had gambled in the past 12 months. Participants were recruited via advertisements on Internet gambling sites (*n* = 2,475) and on gambling-related sites (*n* = 535), such as help services. Participants were also recruited through advertisements on Facebook (*n* = 810) and via Google AdWords (*n* = 288). These recruitment methods were employed to specifically oversample online gamblers. The overall sample was mostly male (77.8%) with a mean age of 42.1 years (*SD* = 14.7).

### Inclusion Criteria

Inclusion criteria were: (a) non-problem or low-risk gamblers based on the Problem Gambling Severity Index (PGSI; Ferris and Wynne, 2001) who reported gambling online on EGMs, sports betting or race betting, or (b) moderate-risk or problem gamblers (also based on the PGSI) who specifically attributed their gambling problem to online gambling on EGMs, sports betting or race betting. The latter criterion was determined by two survey questions. The first question asked respondents which gambling mode of access had contributed most to any problems they may have experienced from their gambling. As we were specifically interested in problems related to online gambling, we excluded anyone who stated that their problems were due to land-based or telephone gambling. The second question asked about the most problematic form of gambling. Ten forms were surveyed (such as instant scratchies, bingo, keno, lotteries). The three most commonly selected forms amongst those who stated that their problems were due to online gambling were: online race betting (*n* = 291), online sports betting (*n* = 181) and online EGM gambling (*n* = 98). All other forms were selected by too few respondents to be included in the following analyses. As the respondents could only select one form (race betting, sports betting or EGM gambling), the MR/PG groups are mutually exclusive.

### Measures

#### Problem Gambling

All respondents completed the 9-item PGSI (Ferris and Wynne, 2001), which is the most widely used and recommended measure of problem gambling severity in Australia (Problem Gambling Research and Treatment Centre, 2011). Response options were “never” (0), “sometimes” (1), “most of the time” (2) and “almost always” (3). Based on their total score, respondents were then categorized into non-problem gambler (0), low risk gambler (1–2), moderate risk gambler (3–7) and problem gambler (8–27). Cronbach’s alpha for the PGSI in this sample was 0.93.

#### Demographics

Respondents completed questions related to their: gender (male/female), age (in years), education (recoded into those with or without a tertiary degree), income, work status (recoded into those working and not working, such as those on a pension, retired, unemployed), country of birth (Australia or other), and main language spoken at home (English or other).

#### Gambling Behavior

Respondents completed questions related to: frequency of engagement on each of ten forms of gambling over the last 12 months (which was also used to calculate a variable that determines how many of the different forms they engage in), the percentage of engagement in each form that was done online, self-reported gambler status (professional, semi-professional, amateur), alcohol use when gambling (no vs. at least sometimes), and illicit drug use when gambling (no vs. at least sometimes). Those who stated that they had gambling-related problems were also asked whether they thought they needed help in relation to their gambling (no or yes) and whether they had ever sought any of 10 types of help in relation to their gambling (recoded into no help-seeking vs. yes to any combination of the 10 types of help).

#### Psychological Variables

Respondents completed the Kessler 6 (K6; Kessler et al., 2002). For each of the K6 items covering symptoms of nervousness, hopelessness, restlessness, depression, worthlessness, and effort, the response options were: ‘none of the time’ (0), ‘a little of the time’ (1), ‘some of the time’ (2), ‘most of the time’ (3) and ‘all of the time’ (4). A sum of the scores on all six items was calculated to give an index of psychological distress. Despite the widespread use of the K6, no clear optimal scoring standards are available (Kessler et al., 2010). The most commonly used thresholds based on validation studies were therefore used: scores of 0–12 indicating no distress, 13+ indicating mild to high levels of distress, and raw scores were also analyzed. Respondents were also asked about their attitudes toward gambling, on a scale from “the harms far outweigh the benefits” to “the benefits far outweigh the harms,” with lower scores indicating a more negative attitude toward gambling.

### Data Analysis

We conducted two major sets of analyses. First we compared the moderate-risk/problem gamblers whose problems reportedly stemmed from each online form (EGMs, sports betting, race betting) to non-problem/low-risk gamblers who engaged in that form online. Hereafter, we refer to the former group as problematic online gamblers and the latter group as non-problematic online gamblers.

The second set of analyses compared three groups of respondents: those whose problems reportedly stemmed from online EGMs, online race betting and online sports betting. All gamblers in these groups were moderate risk or problem gamblers and had nominated that online gambling on that form was responsible for their gambling-related problems.

Both sets of analyses followed the same structure: the relevant groups were compared on demographic, gambling behavior and psychological variables using bivariate, pairwise analyses. These were conducted using chi-square tests of independence (with pairwise tests of proportions where required) for categorical variables, or with one-way ANOVA (with Tukey pairwise comparisons where required) for continuous variables. For gambling frequency and percentage of gambling done online, Mann–Whitney *U*-tests were conducted. Effect sizes are reported for statistically significant results.

Given that discriminatory power can be shared between two or more independent variables, binary logistic regressions predicting problem gambling status were also conducted, to determine which of the significant variables from the bivariate analyses remained significant when controlling for the other variables. An alpha of 0.05 was used throughout.

In terms of missing data, the income question included an option to not disclose this information. This variable was considered in the bivariate analyses, but not the logistic regressions. We did also perform regressions including income, noting that it made very little difference to the results. Therefore, we have opted to report the results with income excluded. The K6 was also excluded from the regression results, as it was highly correlated (>0.6) with the PGSI (which was included as a predictor in the first comparisons, and was the factor that differentiated the groups in the latter comparisons). Tolerance checks were also conducted on the regression models and tolerance was >0.4 for all variables in all models, once K6 was excluded.

We also explored other possible analyses, including multinomial logistic regression models, where all predictors included in the binomial logistic regression models reported in **Tables 8–10** were included, in order to explore potential concerns around Type I error and predictor choice. The results were very similar to those from the binomial logistic regression models. We have opted to retain the binomial results because we see the comparisons between the groups as separate analyses that require separate predictors, and have thus modeled each comparison separately.

### Ethics Statement

This study was carried out in accordance with the recommendations of the National Statement on the Ethical Conduct of Research Involving Humans, and was reviewed and approved by Southern Cross University Human Research Ethics Committee. All subjects gave written informed consent in accordance with the Declaration of Helsinki. Because of the sensitive nature of the survey and the vulnerability of the sample, an informed consent preamble warned that some questions may have been confronting and challenging for some respondents, assured respondents of confidentiality and anonymity, and advised that respondents could withdraw their participation at any time. The survey contained contact details for telephone and online gambling help services.

## Results

### Comparisons between Non-problematic Online EGM Gamblers and Problematic Online EGM Gamblers

#### Bivariate Analyses

Compared to non-problematic online EGM gamblers, problematic online EGM gamblers had significantly lower incomes. They gambled on EGMs more frequently, and were significantly more likely to use alcohol or illicit drugs at least some of the time when gambling. They were significantly more likely be experiencing psychological distress, and to have significantly more negative attitudes toward gambling (**Table 1**).

**Table 1:** Bivariate analyses comparing non-problematic and problematic online EGM gamblers.

Variable	Non-problematic online EGM gamblers (*n* = 64)	Problematic online EGM gamblers (*n* = 98)	Inferential statistics
**Demographics**			
Gender (% male)	68.8	71.4	χ^2^(1, *N* = 162) = 0.13, *p* = 0.715
Age (Mean/SD)	39.6 (15.3)	36.8 (12.7)	*F*(1,160) = 1.59, *p* = 0.209
Education (% with degree)	34.4^∗^	15.3	χ^2^(1, *N* = 162) = 7.99, *p* = 0.005, Φ = 0.22
Work status (% working)	68.8	76.5	χ^2^(1, *N* = 162) = 1.20, *p* = 0.273
Income ($000’s, Mean, *SD*)	86.1^∗^ (42.7)	65.8 (42.7)	*F*(1,144) = 7.72, *p* = .006, η^2^ = 0.05
Country of birth (% Australia)	75.0	83.7	χ^2^(1, *N* = 162) = 1.84, *p* = 0.175
Main language spoken at home (% English)	85.9	88.8	χ^2^(1, *N* = 162) = 0.29, *p* = 0.591
**Gambling behaviour variables**			
Frequency of gambling on EGMs in last 12 months (median)	2.0	4.0^∗^	*Mann–Whitney U* = 1897.5, *p* < 0.001
Percentage of EGM gambling online in last 12 months (median)	50	60	*Mann–Whitney U* = 576.5, *p* = 0.070
Number of forms in last 12 months (mean, *SD*)	5.2 (2.0)	5.7 (1.8)	*F*(1,160) = 2.55, *p* = 0.112
Gambler status			χ^2^(2, *N* = 162) = 1.01, *p* = 0.605
Professional	0.0	1.0	
Semi-professional	9.4	12.2	
Amateur (%)	90.6	86.7	
Alcohol use when gambling (% at least sometimes)	57.8	77.6^∗^	χ^2^(1, *N* = 162) = 7.15, *p* = 0.007, Φ = 0.21
Drug use when gambling (% at least sometimes)	6.3	23.5^∗^	χ^2^(1, *N* = 162) = 8.27, *p* = 0.004, Φ = 0.23
**Psychological variables**			
Kessler 6 (grouped, % high psychological distress)	0.0	21.4^∗^	χ^2^(1, *N* = 162) = 15.76, *p* < 0.001, Φ = 0.31
Kessler 6 score (mean, *SD*)	1.8 (3.1)	7.4^∗^ (6.3)	*F*(1,160) = 43.11, *p* < 0.001, η^2^ = 0.21
Attitudes toward gambling (mean, *SD*)	1.2^∗^ (1.3)	0.7 (1.0)	*F*(1,160) = 6.03, *p* = 0.015, η^2^ = 0.04

*Asterisks indicate a significantly higher mean, proportion or median in that row.*

#### Multivariate Analyses

All of the significant variables from the bivariate analyses were included in a multivariate logistic regression, with the exception of income and Kessler 6. Lowest tolerance between the variables was 0.85, indicating no multicollinearity problems. The overall model was significant χ^2^(5, *N* = 162) = 36.68, *p* < 0.001 and correctly classified 70.5% of cases overall.

The results were similar to the bivariate analyses, with the exception that the gambling attitudes variable was no longer significant when controlling for the other variables in the model (**Table 2**).

**Table 2:** Logistic regression predicting non-problematic online EGM gamblers compared to problematic online EGM gamblers.

Variable	*B*	*SE*	Wald	*p*	OR	95% C.I. for OR	
						Lower	Upper
**Education (ref = tertiary)**	**1.03**	**0.44**	**5.50**	**0.019**	**2.80**	**1.18**	**6.63**
**EGM gambling frequency**	**0.39**	**0.12**	**11.10**	**0.001**	**1.47**	**1.17**	**1.84**
**Alcohol use while gambling (ref = no)**	**0.97**	**0.40**	**5.81**	**0.016**	**2.63**	**1.20**	**5.79**
**Drug use while gambling (ref = no)**	**1.21**	**0.62**	**3.89**	**0.049**	**3.37**	**1.01**	**11.24**
Gambling Attitudes	-0.25	0.16	2.41	0.120	0.78	0.57	1.07
*Constant*	*-2.16*	*0.64*	*11.35*	*0.001*	*0.11*		

*Non-problematic online EGM gamblers coded as 0, problematic online EGM gamblers coded as 1. Therefore, positive coefficients indicate that a higher score on that variable is associated with problematic online EGM gamblers. Bold text indicates a significant predictor. Income was not included due to missing data, and Kessler 6 was not included due to a high correlation with the PGSI.*

### Comparisons between Non-problematic Online Sport Gamblers and Problematic Online Sports Bettors

#### Bivariate Analyses

Compared to non-problematic online sports bettors, problematic online sports bettors were significantly more likely to be male, younger, have a lower income, be born outside of Australia, and speak a language other than English as their main language at home. They gambled on sports more frequently, but did less of their sports betting online, and were significantly more likely to consider themselves to be semi-professional gamblers. They were significantly more likely to use illicit drugs at least some of the time when gambling, to be experiencing psychological distress, and to have more negative attitudes toward gambling (**Table 3**).

**Table 3:** Bivariate analyses comparing non-problematic and problematic online sports bettors.

Variable	Non-problematic online sports gamblers (*n* = 1213)	Problematic online sports gamblers (*n* = 181)	Inferential statistics
**Demographics**			
Gender (% male)	90.4	98.3^∗^	χ^2^(1, *N* = 1394) = 12.61, *p* < 0.001, Φ = 0.10
Age (Mean/*SD*)	41.3^∗^ (14.0)	31.1 (9.8)	*F*(1,1392) = 88.58, *p* < 0.001, η^2^ = 0.06
Education (% with degree)	42.9	44.8	χ^2^(1, *N* = 1394) = 0.23, *p* = 0.633
Work status (% working)	79.2	77.3	χ^2^(1, *N* = 1394) = 0.33, *p* = 0.563
Income ($000’s, Mean, *SD*)	91.9^∗^ (44.6)	82.3 (49.4)	*F*(1,1254) = 6.33, *p* = 0.012, η^2^ = 0.01
Country of birth (% Australia)	84.4^∗^	76.2	χ^2^(1, *N* = 1394) = 7.59, *p* = 0.006, Φ = 0.07
Main language spoken at home (% English)	93.1^∗^	77.9	χ^2^(1, *N* = 1394) = 44.75, *p* < 0.001, Φ = 0.18
**Gambling behaviour variables**			
Frequency of gambling on sports in last 12 months (median)	4.0	6.0^∗^	*Mann–Whitney U* = 56829, *p* < 0.001
Percentage of sports gambling online in last 12 months (median)	100^∗^	98.0	*Mann–Whitney U* = 92808.5, *p* = 0.012
Number of forms in last 12 months (mean, *SD*)	4.5 (1.7)	4.6 (2.2)	*F*(1,1392) = 0.92, *p* = 0.337
Gambler status			χ^2^(2, *N* = 1394) = 44.75, *p* < 0.001, Φ = 0.18
Professional	2.3	3.3	
Semi-professional	8.0	16.0^∗^	
Amateur (%)	89.7^∗^	80.7	
Alcohol use when gambling (% at least sometimes)	67.7	64.1	χ^2^(1, *N* = 1394) = 0.92, *p* = 0.336
Drug use when gambling (% at least sometimes)	3.5	8.8^∗^	χ^2^(1, *N* = 1394) = 10.89, *p* = 0.001, Φ = 0.09
**Psychological variables**			
Kessler 6 (grouped, % high psychological distress)	0.7	12.7^∗^	χ^2^(1, *N* = 1394) = 105.14, *p* < 0.001, Φ = 0.28
Kessler 6 score (mean, *SD*)	1.7 (2.6)	6.4^∗^ (5.3)	*F*(1,1392) = 351.27, *p* < 0.001, η^2^ = 0.20
Attitudes toward gambling (mean, *SD*)	1.4^∗^ (1.2)	1.1 (1.0)	*F*(1,1392) = 9.56, *p* = 0.002, η^2^ = 0.01

*Asterisks indicate a significantly higher mean, proportion or median in that row.*

#### Multivariate Analyses

All of the significant variables from the bivariate analyses were included in a multivariate logistic regression. Lowest tolerance between the variables was 0.89, indicating no multicollinearity problems. The overall model was significant χ^2^(10, *N* = 1394) = 243.25, *p* < 0.001 and correctly predicted 73.0% of cases overall.

The results were relatively similar to the bivariate analyses, although percentage of sports betting conducted online, self-reported professional gambling status and drug use when gambling were no longer significant (**Table 4**). Problematic online sports bettors were significantly more likely to be male, younger, born in a country other than Australia, speak a language other than English at home, gamble on sports more frequently and have a more negative attitude toward gambling.

**Table 4:** Logistic regression predicting non-problematic online sports bettors compared to problematic online sports bettors.

Variable	*B*	*SE*	Wald	*p*	OR	95% C.I. for OR	
						Lower	Upper
**Gender (ref = female)**	**1.35**	**0.63**	**4.61**	**0.032**	**3.85**	**1.13**	**13.20**
**Age (in years)**	**-0.07**	**0.01**	**59.71**	**<0.001**	**0.93**	**0.92**	**0.95**
**Country of birth (ref = not Australia)**	**-0.57**	**0.25**	**5.27**	**0.022**	**0.57**	**0.35**	**0.92**
**Main language at home (ref = language other than English)**	**-0.66**	**0.28**	**5.67**	**0.017**	**0.52**	**0.30**	**0.89**
**Sports betting frequency**	**0.65**	**0.08**	**69.92**	**<0.001**	**1.91**	**1.64**	**2.23**
% sports betting online	-0.00	0.01	0.04	0.851	1.00	0.99	1.01
Professional status (ref = amateur)			1.58	0.453			
Semi-professional	0.24	0.58	0.17	0.682	1.27	0.41	3.99
Professional	0.50	0.55	0.84	0.358	1.65	0.57	4.84
Drug use while gambling (ref = no)	0.15	0.38	0.16	0.689	1.17	0.55	2.47
**Gambling attitudes**	**-0.33**	**0.09**	**14.53**	**<0.001**	**0.72**	**0.60**	**0.85**
*Constant*	*-2.98*	*1.063*	*7.86*	*0.005*	*0.05*		

*Non-problematic online sports bettors coded as 0, problematic online sports bettors coded as 1. Therefore, positive coefficients indicate that a higher score on that variable is associated with problematic online sports bettors. Bold text indicates a significant predictor. Income was not included due to missing data, and Kessler 6 was not included due to a high correlation with the PGSI.*

### Comparisons between Non-problematic Online Race Bettors and Problematic Online Race Bettors

#### Bivariate Analyses

Compared to non-problematic online race bettors, problematic online race bettors were significantly more likely to be male, younger, less likely to have a degree, and more likely to have a lower income, be born in Australia, and to speak a language other than English at home. They gambled on races more frequently, did less of their race betting online, and were significantly more likely to gamble on more forms of gambling. They were significantly more likely to rate themselves as a semi-professional gambler, and to use drugs at least sometimes when gambling. They were significantly more likely be experiencing psychological distress, and to have more negative attitudes toward gambling (**Table 5**).

**Table 5:** Bivariate analyses comparing non-problematic and problematic online race bettors.

Variable	Non-problematic online race bettors (*n* = 1145)	Problematic online race bettors (*n* = 291)	Inferential statistics
**Demographics**			
Gender (% male)	88.8	96.2^∗^	χ^2^(1, *N* = 1436) = 14.53, *p* < 0.001, Φ = 0.10
Age (Mean/SD)	43.5^∗^ (14.4)	39.0 (12.8)	*F*(1,1434) = 23.82, *p* < 0.001, η^2^ = 0.02
Education (% with degree)	41.4^∗^	35.4	χ^2^(1, *N* = 1436) = 3.48, *p* = 0.062, Φ = 0.15
Work status (% working)	77.6	81.1	χ^2^(1, *N* = 1436) = 1.64, *p* = 0.201
Income ($000’s, Mean, *SD*)	91.4^∗^ (44.8)	84.4 (43.7)	*F*(1,1310) = 5.33, *p* = 0.021, η^2^ = 0.01
Country of birth (% Australia)	85.0	89.7^∗^	χ^2^(1, *N* = 1436) = 4.26, *p* = 0.039, Φ = 0.05
Main language spoken at home (% English)	94.5^∗^	89.7	χ^2^(1, *N* = 1436) = 8.85, *p* = 0.003, Φ = 0.08
**Gambling behavior variables**			
Frequency of gambling on races in last 12 months (median)	4.0	6.0^∗^	*Mann–Whitney U* = 90515.5, *p* < 0.001
Percentage of race betting online in last 12 months (median)	95.0^∗^	90.0	*Mann–Whitney U* = 141509, *p* = 0.012
Number of forms in last 12 months (mean, *SD*)	4.5 (1.7)	4.9^∗^ (1.8)	*F*(1,1434) = 13.80, *p* < 0.001, η^2^ = 0.01
Gambler status			χ^2^(2, *N* = 1436) = 7.58, *p* = 0.023, Φ = 0.07
Professional	2.4	0.7	
Semi-professional	8.4	12.4^∗^	
Amateur (%)	89.2	86.9	
Alcohol use when gambling (% at least sometimes)	67.6	73.2	χ^2^(1, *N* = 1436) = 3.38, *p* = 0.066
Drug use when gambling (% at least sometimes)	3.0	9.3^∗^	χ^2^(1, *N* = 1436) = 22.71, *p* < 0.001, Φ = 0.13
**Psychological variables**			
Kessler 6 (grouped, % high psychological distress)	0.5	11.7^∗^	χ^2^(1, *N* = 1436) = 106.71, *p* < 0.001, Φ = 0.27
Kessler 6 score (mean, *SD*)	1.6 (2.5)	5.1^∗^ (5.0)	*F*(1,1434) = 284.00, *p* < 0.001, η^2^ = 0.17
Attitudes toward gambling (mean, *SD*)	1.4^∗^ (1.2)	1.0 (1.0)	*F*(1,1434) = 30.04, *p* < 0.001, η^2^ = 0.02

*Asterisks indicate a significantly higher mean, proportion or median in that row.*

#### Multivariate Analyses

All of the significant variables from the bivariate analyses were included in a multivariate logistic regression. Lowest tolerance between the variables was 0.83, indicating no multicollinearity problems. The overall model was significant χ^2^(12, *N* = 1434) = 275.49, *p* < 0.001 and correctly predicted 75.3% of cases overall.

The results were relatively similar to the bivariate analyses, although education, country of birth and percentage of race betting conducted online were no longer significant (**Table 6**). Problematic online race bettors were significantly more likely to be male, younger, speak a language other than English at home, gamble on races more frequently, engage in more forms of gambling, self-report as a semi-professional or professional gambler, use drugs whilst gambling, and have a more negative attitude toward gambling.

**Table 6:** Logistic regression predicting non-problematic online race bettors compared to problematic online race bettors.

	*B*	*SE*	Wald	Sig.	OR	95% C.I. for OR	
						Lower	Upper
**Gender (ref = female)**	**0.81**	**0.36**	**4.90**	**0.027**	**2.24**	**1.10**	**4.57**
**Age (in years)**	**-0.04**	**0.01**	**33.33**	**<0.001**	**0.97**	**0.96**	**0.98**
Education (ref = tertiary)	0.15	0.16	0.86	0.355	1.16	0.85	1.57
Country of birth (ref = not Australia)	0.35	0.25	2.01	0.156	1.42	0.87	2.31
**Main language at home (ref = language other than English)**	**-0.74**	**0.29**	**6.57**	**0.010**	**0.48**	**0.27**	**0.84**
**Race betting frequency**	**0.60**	**0.06**	**116.49**	**<0.001**	**1.82**	**1.64**	**2.03**
% of race betting online	0.02	0.00	3.66	0.056	1.01	1.00	1.01
**Number of forms engaged in**	**0.11**	**0.05**	**5.36**	**0.021**	**1.11**	**1.02**	**1.21**
**Professional status (ref = amateur)**			**6.48**	**0.039**			
**Semi-professional**	**1.63**	**0.78**	**4.34**	**0.037**	**5.09**	**1.10**	**23.54**
**Professional**	**1.84**	**0.76**	**5.91**	**0.015**	**6.30**	**1.43**	**27.81**
**Drug use while gambling (ref = no)**	**0.70**	**0.33**	**4.65**	**0.031**	**2.02**	**1.07**	**3.82**
**Gambling attitudes**	**-0.44**	**0.07**	**36.65**	**<0.001**	**0.64**	**0.56**	**0.74**
*Constant*	*-5.44*	*1.04*	*27.59*	*<0.001*	*0.01*		

*Non-problematic online race bettors coded as 0, problematic online race bettors coded as 1. Therefore, positive coefficients indicate that a higher score on that variable is associated with problem online race bettors. Bold text indicates a significant predictor. Income was not included due to missing data, and Kessler 6 was not included due to a high correlation with the PGSI.*

### Comparisons between Problematic Online EGM Gamblers, Problematic Online Race Bettors and Problematic Online Sports Bettors

The following analyses identify distinguishing characteristics between problematic online EGM gamblers, problematic online race bettors and problematic online sports bettors.

#### Bivariate Analyses

##### Demographics

Problematic online sports bettors and race bettors were significantly more likely to be male, have a tertiary degree and have higher incomes compared to problematic online EGM gamblers. Problematic online sports bettors were significantly younger compared to both problematic online EGM gamblers and problematic online race bettors. Problematic online sports bettors were significantly less likely to be born in Australia, and significantly more likely to speak a language other than English at home compared to problematic online race bettors, with problematic online EGM gamblers not significantly different to either of the other groups on these variables (**Table 7**).

**Table 7:** Descriptive statistics and inferential tests for demographic variables by most problematic online gambling form.

Variable	Problematic online EGM gamblers (*n* = 98)	Problematic online sports bettors (*n* = 181)	Problematic online race bettors (*n* = 291)	Inferential statistics
**Demographics**				
Gender (% male)	71.4a	98.3b	96.2b	χ^2^(2, *N* = 570) = 78.69, *p* < 0.001, Φ = 0.37
Age (Mean/SD)	36.8a (12.7)	31.1b (9.8)	39.0a (12.8)	*F*(2,567) = 25.46, *p* < 0.001, η^2^ = 0.08
Education (% with degree)	15.3a	44.8b	35.4b	χ^2^(2, *N* = 570) = 24.32, *p* < 0.001, Φ = 0.21
Work status (% working)	76.5	77.3	81.1	χ^2^(2, *N* = 570) = 1.43, *p* = 0.489
Income ($000’s, Mean, *SD*)	65.7a (42.4)	81.7b (49.6)	83.8b (44.3)	*F*(2,535) = 5.66, *p* = 0.004, η^2^ = 0.02
Country of birth (% Australia)	83.7a,b	76.2b	89.7a	χ^2^(2, *N* = 570) = 15.36, *p* < 0.001, Φ = 0.16
Main language spoken at home (% English)	88.8a,b	77.9b	89.7a	χ^2^(2, *N* = 570) = 13.59, *p* = 0.001, Φ = 0.15
**Gambling behavior**				
Number of forms in last 12 months (mean, *SD*)	5.7a (1.8)	4.6b (2.2)	4.9b (1.8)	*F*(2,567) = 9.84, *p* < 0.001, η^2^ = 0.03
Gambler status				χ^2^(2, *N* = 570) = 6.92, *p* = 0.140
Professional	1.0a	3.3a	0.7a	
Semi-professional	12.2a	16.0a	12.4a	
Amateur (%)	86.7a	80.7a	86.9a	
PGSI (mean, *SD*)	9.5a (6.0)	8.3ab (4.6)	7.4b (4.6)	*F*(2,567) = 7.55, *p* = 0.001, η^2^ = 0.03
Alcohol use when gambling (% at least sometimes)	77.6a	64.1b	73.2a,b	χ^2^(2, *N* = 570) = 6.93, *p* = 0.031, Φ = 0.11
Drug use when gambling (% at least sometimes)	23.5a	8.8b	9.3b	χ^2^(2, *N* = 570) = 16.36, *p* < 0.001, Φ = 0.17
**Psychological variables**				
Kessler 6 (grouped, % high psychological distress)	21.4a	12.7ab	11.7b	χ^2^(2, *N* = 570) = 6.11, *p* = 0.047, Φ = 0.10
Kessler 6 score (mean, *SD*)	7.4a (6.3)	6.4ab (5.3)	5.1b (5.0)	*F*(2,567) = 8.24, *p* < 0.001, η^2^ = 0.03
Attitudes toward gambling (mean, *SD*)	-1.27a (0.96)	-0.90b (0.99)	-1.00a,b (1.04)	*F*(2,567) = 4.33, *p* = 0.014, η^2^ = 0.02
Problems emerged after you first gambled online (% after)	41.8a	64.4b	51.6a	χ^2^(2, *N* = 540) = 12.97, *p* = 0.002, Φ = 0.16
Thought you needed help in relation to your gambling (% yes)	54.1a	35.9b	44.0a,b	χ^2^(2, *N* = 570) = 8.72, *p* = 0.013, Φ = 0.12
Ever sought help (% yes)	46.9a	31.5b	27.5b	χ^2^(2, *N* = 570) = 12.77, *p* = 0.002, Φ = 0.15

*Subscripts indicate pairwise comparisons (tests of proportions for all variables apart from age, which are Tukey tests). Groups with the same letter are not significantly different from each other, and groups with two subscripts are not significantly different from either of the other groups. For example, for country of birth, a significantly higher proportion of problematic online race bettors was born in Australia compared to problematic online sports bettors (different subscripts), while problematic online EGM gamblers are not significantly different from either group. If no subscripts are present, no significant differences were observed.*

##### Gambling behavior

Problematic online EGM gamblers were significantly more likely to participate in more forms of gambling compared to both problematic online sports bettors and race bettors, and were significantly more likely to use illicit drugs when gambling compared to both of these groups. Problematic online EGM gamblers were also significantly more likely to drink alcohol at least sometimes when gambling compared to problematic online sports bettors. No significant differences were observed in terms of self-rated professional gambling status. Problematic online EGM gamblers had significantly higher PGSI scores compared to problematic online race bettors, with problematic online sports bettors not significantly different to either group.

##### Psychological variables

Problematic online EGM gamblers were significantly more likely to be experiencing high psychological distress compared to problematic online race bettors, and also to have higher K6 scores, with problematic online sports bettors being not significantly different to either group. Problematic online EGM gamblers were significantly more likely to agree that gambling harms outweighed benefits compared to problematic online sports bettors, with problematic online race bettors not significantly different to either group (**Table 7**).

Problematic online sports bettors were significantly more likely to state that their problems emerged after they first gambled online. Problematic online EGM gamblers were significantly more likely to think they needed help in relation to their gambling compared to problematic online sports bettors, and were significantly more likely to have sought help compared to problematic online sports and race bettors.

#### Multivariate Analyses

In order to account for any overlap between the bivariate analyses comparing problematic online EGM gamblers, sports bettors and race bettors, we conducted three separate binary logistic regressions. The first compared problematic online EGM gamblers to problematic online sports bettors; the second compared problematic online EGM gamblers to problematic online race bettors; and the third compared problematic online sports bettors to problematic online race bettors.

The predictors included in each regression were the variables that showed significant differences for each comparison. For example, attitudes toward gambling differed significantly between problematic online EGM gamblers and problematic online sports bettors, and was thus included in the first regression. However, problematic online race bettors did not differ significantly to either of the other groups, and thus this variable was not included in either of the other regression analyses.

For the regression comparing problematic online EGM gamblers and problematic online sports bettors, gender was an issue, as only three problematic online sports bettors were female, and thus was virtually constant for that group. Gender was therefore dropped from the regression.

##### Logistic regression: problematic online EGM gamblers vs. problematic online sports bettors

The model was significant [χ^2^(9, *N* = 279) = 66.52, *p* < 0.001] and correctly predicted 68.8% of the sample. Compared to problematic online EGM gamblers, problematic online sports bettors were significantly younger, more educated, and engaged in significantly fewer forms of gambling (**Table 8**).

**Table 8:** Multivariate logistic regression results predicting problematic online EGM gamblers vs. problematic online sports bettors.

Variable	*B*	*SE*	Wald	*p*	OR	95% C.I. for OR	
						Lower	Upper
**Age in years**	**-0.05**	**0.01**	**14.59**	**<0.001**	**0.95**	**0.92**	**0.97**
**Education (ref = non-tertiary)**	**1.28**	**0.35**	**13.62**	**<0.001**	**3.59**	**1.82**	**7.08**
**Number of forms engaged in**	**-0.27**	**0.08**	**13.24**	**<0.001**	**0.76**	**0.66**	**0.88**
Professional status (ref = amateur)			0.52	0.771			
Semi-professional	0.28	0.42	0.45	0.503	1.32	0.58	3.01
Professional	0.35	1.13	0.10	0.757	1.42	0.16	12.96
PGSI score	-0.01	0.03	0.10	0.747	0.99	0.93	1.06
Thought they needed help (ref = no)	-0.14	0.36	0.15	0.697	0.87	0.43	1.75
Sought help (ref = no)	-0.43	0.33	1.73	0.188	0.65	0.34	1.24
Gambling attitudes	0.26	0.17	2.35	0.125	1.29	0.93	1.80
*Constant*	3.55	0.83	18.43	<0.001	34.72		

*Problematic online EGM coded as 0, problematic online sports bettor coded as 1. Therefore, positive coefficients indicate that a higher score on that variable is associated with online sports bettors. Bold text indicates a significant predictor.*

##### Logistic regression: problematic online EGM gamblers vs. problematic online race bettors

The model was significant [χ^2^(6, *N* = 389) = 88.52, *p* < 0.001] and correctly predicted 75.8% of the sample. Compared to problematic online EGM gamblers, problematic online race bettors were significantly more likely to be male, have higher education, gamble on fewer forms, and were significantly less likely to use illicit drugs when gambling (**Table 9**).

**Table 9:** Multivariate logistic regression results predicting problematic online EGM vs. problematic online race bettors.

Variable	*B*	*SE*	Wald	*p*	OR	95% C.I. for OR	
						Lower	Upper
**Gender (ref = female)**	**2.55**	**0.42**	**36.36**	**<0.001**	**12.84**	**5.60**	**29.42**
**Education (ref = non-tertiary)**	**0.98**	**0.34**	**8.36**	**0.004**	**2.67**	**1.37**	**5.21**
**Number of forms engaged in**	**-0.28**	**0.08**	**14.00**	**<0.001**	**0.75**	**0.65**	**0.87**
PGSI score	-0.04	0.03	2.25	0.134	0.96	0.91	1.01
**Drug use while gambling (ref = no)**	**-0.79**	**0.36**	**5.00**	**0.025**	**0.45**	**0.23**	**0.91**
Sought help (ref = no)	-0.49	0.30	2.58	0.108	0.62	0.34	1.12
*Constant*	0.78	0.55	1.99	0.158	2.18		

*Problematic online EGM coded as 0, problematic online race bettor coded as 1. Therefore, positive coefficients indicate that a higher score on that variable is associated with online race bettors. Bold text indicates a significant predictor.*

##### Logistic regression – problematic online sports bettors vs. problematic online race bettors

The model was significant [χ^2^(3, *N* = 472) = 73.01, *p* < 0.001] and correctly predicted 65.9% of the sample. Compared to problematic online sports bettors, problematic online race bettors were significantly older and significantly more likely to be born in Australia (**Table 10**).

**Table 10:** Multivariate logistic regression results predicting problematic online sports bettors vs. problematic online race bettors.

Variable	*B*	*SE*	Wald	*p*	OR	95% C.I. for OR	
						Lower	Upper
**Age in years**	**0.06**	**0.01**	**43.45**	**<0.001**	**1.07**	**1.05**	**1.09**
**Country of birth (ref = not Australia)**	**1.16**	**0.30**	**14.72**	**<0.001**	**3.19**	**1.76**	**5.77**
Main language spoken at home (ref = not English)	0.46	0.29	2.43	0.119	1.58	0.89	2.81
Constant	-3.10	0.48	41.44	<0.001	0.05		

*Problematic online sports bettors coded as 0, problematic online race bettors coded as 1. Therefore, positive coefficients indicate that a higher score on that variable is associated with online race bettors. Bold text indicates a significant predictor.*

## Discussion

This paper is the first to our knowledge to identify risk factors specific to problematic gambling on three popular forms of online gambling – EGMs, race betting and sports betting. While previous studies have identified risk factors for problem gamblers who engage in Internet gambling (e.g., McBride and Derevensky, 2009; Wood and Williams, 2009; Potenza et al., 2011; Hing et al., 2016e), these analyses have not considered whether or not their gambling problems are related to specific gambling forms and to engaging with them online. Using that approach may cloud results, given that online problem gamblers are often mixed-mode gamblers who gamble on multiple forms (Wardle et al., 2011; Gainsbury et al., 2015a). The current paper sought to provide a more accurate assessment of these risk factors by considering only individuals whose gambling problems reportedly stemmed from online gambling and from the specified gambling form.

In relation to online EGM gambling, only a few risk factors emerged that distinguished problematic from non-problematic players. Not surprisingly, more frequent online EGM gambling increased the risk of gambling problems, which aligns with findings from risk curve analyses based on several large representative datasets in Canada (Currie et al., 2006, 2008). Those analyses found that the risk of gambling problems increases steadily with frequency of EGM gambling and with the percentage of income spent on gambling. The latter association may also explain why lower income was also a risk factor for the problematic online EGM gamblers in our study. Other distinguishing risk factors were higher likelihood of using alcohol or illicit drugs while gambling, reflecting the greater prevalence of substance use amongst problem gamblers generally (Welte et al., 2004; Blanco et al., 2006; Dannon et al., 2006; Petry, 2007; Castrén et al., 2013). Of potential concern is that being able to gamble online in private may increase the ease of substance use while gambling, which may undermine rational decision-making. However, further research is needed to ascertain whether substance use amongst problem gamblers is more frequently associated with online compared to land-based EGM gambling. Of particular note is that nearly one-quarter of problematic online EGM gamblers reported at least sometimes using illicit drugs while gambling. Thus, the solitary and private character of online EGM play may facilitate long continuous sessions, in which alcohol and drugs are more likely to be consumed. The convenient access to internet gambling at any time of the day facilitates intense use among other work and familial commitments, and presumably helps to conceal the activity from significant others (Gainsbury et al., 2015b). These features would reasonably contribute to greater potential for the development of problems. Future research could profitably focus on comparing the timing and duration of online play sessions between problem and recreational online gamblers.

Our bivariate analyses indicated that problematic online EGM gamblers were significantly more likely to be experiencing psychological distress, compared to non-problematic online EGM players. This result parallels findings for problem gamblers who play land-based EGMs, and who frequently report doing so to escape negative mood states (Blaszczynski and Nower, 2002; Thomas et al., 2009; Nower and Blaszczynski, 2010). Individuals motivated to alleviate psychological distress may find online EGM gambling to be a more attractive escape mechanism, as it is more convenient and less socially demanding than attending a physical venue. The privacy and lack of distractions or interruptions provided by online EGM gambling is likely to contribute to immersion and dissociation – cognitive states thought be associated with ‘escape oriented’ problem gamblers – and which facilitate excessive gambling (Griffiths and Parke, 2002; Griffiths, 2003; Monaghan, 2009; Corney and Davis, 2010). Additional research is needed to test this proposition.

The above findings suggest that interventions for problem online EGM players need to discourage frequent gambling on this activity and substance use while gambling. In domestic venues, steps might conceivably be taken by staff to monitor and intervene with patrons displaying these characteristics. However, in the case of online EGM providers, such measures are far more difficult to implement or enforce. High rates of psychological distress and higher PGSI scores in this cohort indicate that interventions involving professional treatment may be the most appropriate to address these underlying issues. In this study, a result of note is that the problematic online EGM gamblers were more likely to think they needed help for their gambling and to have sought help, than their race betting and sports betting counterparts. This increased ability of online EGM players to recognize they have a problem is understandable given the negative mood states more commonly associated with this form of play. The characteristics of problematic online EGM gamblers also suggest that interventions should target both genders and age groups from young to middle aged adults, and take into account the lower educational and income levels of this group. Interventions should also challenge beliefs that one can earn money from gambling, and discourage gambling on multiple gambling activities. Importantly, three-fifths of this cohort had gambling problems before gambling online. Therefore it should be recognized that for most, internet gambling provides a mechanism to sustain a developing dependence, rather than necessarily representing a ‘gateway’ into problematic use. Nevertheless, current Australian regulations outlawing the provision of online EGMs to Australian residents appear prudent, although they remain easily accessible via offshore sites. Given that problem online EGM gamblers also tend to gamble in venues, interventions should also discourage heavy gambling on land-based forms.

Risk factors identified for problematic online sports betting were very similar to those for problematic online race betting. Compared to their non-problematic counterparts, the problematic online sports and race bettors were more likely to be male, younger, and to speak a language other than English at home. This younger male profile of online bettors with gambling problems has also been identified elsewhere (Hing et al., 2017). Being a young adult male has consistently been identified as a risk factor for problem gambling in general (Johansson et al., 2009; Williams et al., 2012; Hing et al., 2016d). Because online wagering is heavily marketed to this demographic, concerns have been raised that young, male Internet bettors face heightened risks of related gambling problems (Lamont et al., 2011; Milner et al., 2013). Mirroring the comments made above regarding EGM players, much may depend on the patterns of use exhibited by online sports betters. For example, if online betting is done sporadically, in a social context (e.g., watching a game together), then online play may represent no extra risk when compared to venue-based play. On the other hand, if online sports betting facilitates different patterns of use (e.g., solitary betting in extended sessions late at night), then this would provide further evidence that the online product presents a greater risk. At the present time, concerns appear to be justified, given that young men in particular are increasingly seeking treatment for difficulties in controlling their online sports betting (Blaszczynski and Hunt, 2011).

Compared to the non-problematic online bettors, online bettors (both sports and race) with gambling problems were less likely to speak English at home. Problematic online sports bettors were less likely to have been born in Australia, while the opposite was true for problematic online race bettors. These findings imply that the problematic online sports bettors were more likely to be first generation migrants, and their race betting counterparts to be second generation migrants, from non-English speaking countries. This difference may partially reflect the older age of problematic online race bettors than sports bettors in this study. Regardless, these findings are consistent with ethnic minority status being a common risk factor for gambling problems (Raylu and Oei, 2004; Welte et al., 2004). It is possible, however, that the ethnic profiles of problematic online sports bettors and race bettors differ, and this is an avenue for further research.

Behavioral risk factors for online bettors in our sample included more frequent online betting, as also found by LaBrie and Shaffer (2011) in their analysis of online betting data provided by a wagering operator. Both problem and non-problem online betters used the Internet for a very large proportion of their play. Nevertheless, problematic online bettors were more likely to also bet offline than their non-problem counterparts. This may be partially explained by a preference among problem sports betters for in-play betting, which is illegal via the Internet in Australia, although it is legal via telephone and in land-based venues. Several studies have found in-play betting to be associated with gambling problems (Gray et al., 2012; Braverman et al., 2013; LaPlante et al., 2014; Hing et al., 2016e). It may be also explained by the general tendency of problematic gamblers to employ multiple forms and platforms for their gambling (Potenza et al., 2000).

The problematic online bettors also had a greater tendency than their non-problematic counterparts to consider themselves to be semi-professional gamblers, and for race bettors, professional gamblers. Previous research has found high rates of problem gambling amongst self-nominated professional gamblers, raising queries over whether self-identifying as a professional gambler is a common but misguided way to rationalize problem gambling (Hing et al., 2015a, 2016b). Browne et al. (2015) argue that race betters are prone to suffer from ‘delusions of expertise,’ due to intrinsic difficulties in self-assessing skill based on betting returns. The problematic online bettors were also more likely to use illicit drugs when gambling, compared to their non-problematic counterparts, while the problematic sports bettors exhibited higher psychological distress than did those without gambling problems. Given these effects mirror the results discussed above for online EGM play, it suggests that similar concerns regarding increased potential risk may also apply to online sports betting.

The preceding results for problematic online bettors imply that interventions need to particularly target young adult males, discourage frequent betting, in-play betting and illicit drug use while betting, and challenge beliefs that one can easily earn money from betting. Public health messages should be available in a range of community languages, given the ethnic diversity of this cohort. Professional treatment that caters for online sports bettors should be encouraged, given their relatively high PGSI scores and psychological distress. These interventions also need to take into consideration the relatively high educational qualifications and income of this group, and also their typical engagement with multiple gambling activities.

Several limitations of this study must be acknowledged. The non-random sample attained means that results may not generalize to the broader population of problematic online gamblers on each of the three forms examined; and the sample size for EGM gamblers was particularly small. The identification of most problematic gambling mode and most problematic gambling form relied on self-report, and the analyses were unable to take into account multiple modes and forms that might be causing gambling problems for some respondents. Further, the study was cross-sectional. While this was adequate to identify risk factors, causal directions between gambling problems and each risk factor could not be ascertained. Finally, some social desirability bias may be present given the survey relied on self-report about a sensitive and stigmatized issue (Hing et al., 2016a,c).

## Conclusion

This study has identified a range of risk factors for problem and moderate risk gambling on online EGMs, online sports betting and online race betting. Prior studies have generally made comparisons between those who gamble online and those who do not. Because online betters are also likely to gamble more heavily on land-based forms, this has prevented strong inference regarding the likely instrumental role of online betting in contributing to problematic play. That is, to some degree online play may *reflect* greater gambling involvement, rather than necessarily drive the development of problems (Hing et al., 2014a). A strength of this study is that it is the first known analysis of risk factors for online gambling based on most problematic mode and most problematic form of gambling. This has enabled comparisons within the set of individuals who gamble online, between those who nominate online gambling as their most problematic form of play and those who do not. This has provided some insight into the risk factors associated with online play. Our interpretation of these effects is that online platforms represent a risk in providing an accessible, convenient, and private means to continuously access gambling products at any time of the day. The privacy inherent to home internet use facilitates the potential concurrent use of alcohol and drugs. Particularly for online EGM gamblers – who tend to be vulnerable to escape-oriented and dissociative motivations – these represent risk factors for excessive use and associated gambling problems.

The detailed pattern of risks tends to vary with regard to different online gambling forms, particularly for EGM gambling when compared to sports betting and race betting. These differences point to the importance of developing and implementing interventions specifically for each online gambling form that are tailored to the characteristics and behaviors of those most at-risk of gambling problems on each of these activities. The findings suggest that interventions for online EGMs could include: general messages on EGM websites and in social marketing that warn of the risks of gambling while under the influence of substances, that challenge beliefs that one can earn money from gambling, and that discourage gambling on multiple gambling activities; dynamic messaging on EGM websites triggered by high frequency of EGM play; and the availability and promotion of limit setting functions on EGM websites that enable gamblers to better restrict their EGM play. These communications need to be tailored to those most at-risk, and to therefore target both genders, age groups from young to middle aged adults, and those from lower educational and income levels. For sports and race bettors, the findings suggest that interventions such as social marketing and warning messages on betting websites need to particularly target young adult males and be available in a range of community languages. These communications should discourage frequent betting, in-play betting and illicit drug use while betting, and challenge beliefs that one can easily earn money from betting. Frequent betting should also trigger dynamic warning messages, while limit setting functions need to be available and prominently promoted on betting websites. Professional treatment catering for online sports bettors should be available, given their relatively high PGSI scores and psychological distress.

## Author Contributions

NH helped to conceive and design the work, helped to organize the acquisition of data, interpreted the data, drafted the manuscript, approved the final version to be published, and agrees to be accountable for all aspects of the work. AR helped to conceive and design the work, helped to organize the acquisition of data, analyzed the data, critically revised the manuscript for important intellectual content, approved the final version to be published, and agrees to be accountable for all aspects of the work. MB helped with data analysis and interpretation, critically revised the manuscript for important intellectual content, approved the final version to be published, and agrees to be accountable for all aspects of the work.

## Conflict of Interest Statement

The authors declare that the research was conducted in the absence of any commercial or financial relationships that could be construed as a potential conflict of interest.
